# Oral health status among long-term hospitalized adults: a cross sectional study

**DOI:** 10.7717/peerj.423

**Published:** 2014-06-10

**Authors:** Leon Bilder, Nirit Yavnai, Avi Zini

**Affiliations:** 1 Department of Periodontology, School of Graduate Dentistry, Rambam Health Care Campus, Haifa, Israel; 2 Medical Corps, IDF, Israel; 3 Department of Community Dentistry, Hadassah School of Dental Medicine, Hebrew University, Hadassah, Jerusalem, Israel

**Keywords:** Oral health, Institutionalised hospital care, Edentulousness and oral hygiene

## Abstract

**Background.** Many Long-Term Care (LTC) institutionalized patients are the most frail and functionally dependent among the geriatric population and have significant oral health disparities.They often suffer from dental neglect due to limited access to appropriate professional dental care. These patients have chronic health situations and are treated with medications, which increase their risk of oral diseases. Despite the growth in elderly population in Israel, there is insufficient data regarding their oral health status and treatment needs.

**Objective.** To describe the oral health status of the LTC hospitalized adults in a geriatric and psychiatric hospital in Israel.

**Methods.** Data was recorded from LTC hospitalized adults with a physical and/or mental disabilities in a cross-sectional research design, which included general health anamnesis and clinical oral examination. Variables included gender, medicines, oral hygiene (OH), using dentures, number of caries lesions and residual teeth. Univariate analyses included Pearson *χ*^2^ and *t*-test analyses. Multivariate analyses included logistic and linear regressions while the outcome variables were categorical OH index and number of carious cavitations, number of residual teeth and carious teeth percentage.

**Results.** 153 participants were included in the study with a mean age of 65.03 ± 18.67 years. 31.3% of the patients were edentulous, and only 14% had partial or full dentures. Females had a significantly higher number of caries cavitation than males (*P* = 0.044). The number of caries cavitation was higher among patients with poor OH (*P* < 0.001) and when taking Clonazepam (*P* = 0.018). Number of residual teeth was higher in the fair OH group (*P* < 0.001). Carious teeth percentage was higher among the poor OH group (*P* < 0.001).

## Introduction

The World Health Organization (WHO) Global Oral Health Program has emphasized the importance of increasing the awareness of oral health worldwide as a major component of general health and quality of life (Petersen et al., 2005). Oral diseases are the most common chronic illnesses and are a significant public health problem due to their prevalence impact on individuals and on society, and the expense of their treatment (Sheiham, 2005).

A report from the U.S. Surgeon General noted that oral problems (e.g., dental caries periodontal disease, tooth loss, dry mouth, oral cancer, chewing problems, pain or discomfort) in older adult and disabled populations are the most common unmet health needs, and that oral diseases are often related to general health problems (National Institute of Dental and Craniofacial Research, 2000). Inferior oral status can lead to serious health consequences, such as abscesses, pain, bacteremia, septicemia and chronic disease (Berkey & Scannapieco, 2013).

Numerous studies have documented a very poor oral health and limited access to dental care among adults residing in long-term care (LTC) facilities (Wyatt, 2002a; Wyatt, 2002b). These patients have physical limitations, chronic diseases, and the majority require medication, which increase the risk of oral diseases and subsequently elevated susceptibility to a lot of side effects with oral manifestations (Peltola, Vehkalahti & Wuolijoki-Saaristo, 2004; Bharti & Bansal, 2013; Abdollahi, Rahimi & Radfar, 2008). In addition, many of those patients receive nasogastric feeding and/or mechanical ventilation and are at risk for malnutrition, especially those living in institutions (Kaiser et al., 2010). Despite the fact that the vast majority of the elderly are living independently, a minority (estimates of 3–5% in United States, Canada, Israel and Finland) are functionally impaired, requiring long-term nursing (Niessen, 2000; Kozyrskyi, De Coster & St John, 2000; Schmid, 2009; Peltola, Vehkalahti & Wuolijoki-Saaristo, 2004), most LTC patients are considered elderly (Kozyrskyi, De Coster & St John, 2000; Peltola, Vehkalahti & Wuolijoki-Saaristo, 2004).

Few studies have shown an association between malnutrition (Saletti et al., 2005; Chan et al., 2010), dentition status (Abnet et al., 2005; Osterberg et al., 2008), and mortality. The institutionalized and homebound LTC elderly patients are the most frail and functionally dependent among the geriatric population with significant oral health disparities (Isaksson & Söderfeldt, 2007). Dental care for the institutionalized is often limited to emergency care and is not aimed to daily oral care (Peltola, Vehkalahti & Wuolijoki-Saaristo, 2004). Many medically compromised LTC patients might have tube fed or suffer from swallowing difficulties (Matear, 1999). That might be related to oral problems such as denture-related diseases, coated tongue, angular cheilitis and Candida-associated denture stomatitis (Peltola, Vehkalahti & Wuolijoki-Saaristo, 2004; Samaranayake et al., 1995; Isaksson & Söderfeldt, 2007).

There is a lack of in-depth information regarding oral health state of the growing elderly population in Israel. At 1948, when the state of Israel was established, elderly population comprised 3% of the total population, as compared to 2008 with slightly over 10%, while most of the LTC patients are treated at their homes (Schmid, 2009).

The present cross-sectional epidemiological survey was conducted at Herzog Hospital in Jerusalem, which is the third largest hospital in the capital and Israel’s foremost center for geriatric and psychiatric health care. This study was part of an intervention program aimed to prevent and improve oral health among long-term hospitalized adults, and was performed from 2010 to 2011.

The aim of this study was to describe the oral health status of the long-term hospitalized adults at Herzog Hospital, Jerusalem.

## Methods

The study population included long-term care inpatients; with inclusion criteria of being hospitalized for more than 6 months at Hertzog Geriatric and Psychiatric Health Care center in Jerusalem. The vast majority of the hospital’s population comprises of disabled, psychiatric and geriatric hospitalized long term patients from a variety of ages. Patients that were undergoing dialysis, acute psychiatric patients and others defined by a hospital nursing team, as a “temporary patients” were excluded. The study was approved by the Hertzog Hospital Institutional Review Board (#180-10). All surveyed patients were older than 18 years old. These patients were previously diagnosed as having physical and/or mental disabilities with no communications skills due to either cognitive or mental reasons. Usage of nasogastric and mechanical ventilation was recorded.

Pre-test clinical examination was carried out by two dentists who were calibrated. The calibration process included clinical examination of ten patients, while data was recorded separately and then a comparison and a discussion on the findings were done. General health anamneses, including medication usage, were recorded from the hospital’s medical files. Clinical examinations included presence of Angular Cheilitis, mucosal lesion, coronal and root decay, number of residual teeth, Oral Hygiene Index (OHI) and denture status. The patients were examined in their rooms at their bedside. The clinical examinations were carried out under artificial light with a dental mirror and a CPI probe (Martin, Solingen, WHO 973/80, Germany). Teeth were neither dried nor cleaned before the examination. Dental status was recorded for each tooth. A tooth was recorded as present when it was fully or partially visible in the mouth. Dental caries were recorded according to the WHO criteria (World Health Organization, 2013) and no radiographs were taken during the evaluation. It was decided that a decayed tooth was counted as a tooth with clinical observed caries, regardless of the number of caries foci on that tooth. DMFT or any other caries experience index was not conducted. The variable “carious teeth percentages” was calculated as the rate between number of dental caries cavitations and the number of residual teeth in the mouth. A modification of the Silness & Löe plaque index was used for the assessment of oral hygiene (OH) (Silness & Loe, 1964) in six index teeth: 16,12, 24, 36, 32, 44. Each of the four teeth surfaces (buccal, lingual, mesial and distal) was given a score from 0 to 3 (0 = No plaque; 1 = a film of plaque adhering to the free gingival margin and adjacent area of the tooth; 2 = moderate accumulation of soft deposits within the gingival pocket or the tooth and gingival margin; 3 = abundance of soft matter within the gingival pocket and/or on the tooth and gingival margin). Mean score was calculated for every participant. No disclosing solution was used in this modification. In the case of absent tooth, adjacent tooth was examined and recorded. In case the whole sextant was absent, no score was recorded. For further analyses, the OHI index results were dichotomized into two categories and were renamed as the variable “OHI2”: poor OH, score of three = 1, high OH (low OH score, fair OH) = 0. The presence of removable dentures was recorded for each jaw and was dichotomized as a full or partial denture per patient.

The data was collected and inserted into an Excel sheet, and transferred to SPSS version 17.0 for statistical analysis. Pearson Chi-square test was employed for testing the statistical significance of differences between gender, taking medicines (categorical variables), OHI2 and using dentures. Independent *t*-test was employed detecting differences between number of caries cavitations, number of residual teeth and percentage of carious teeth with the categorical variables above. Correlation between numerical variables (age, number of teeth and percentage of carious teeth) was employed using Pearson correlation coefficient. The level of significance was set as *P* < 0.05. Multiple Logistic Regression was conducted to eliminate potential confounders and mediators among all the variables tested with OHI2 as the dependent variable. Variables that showed a significance level of less than *P* < 0.15 in the univariate analyses were included in the regression. Linear regression analysis was conducted when the dependent numerical variables were number of caries cavitations, number of residual teeth and percentage of carious teeth.

## Results

One hundred fifty three LTC hospitalized individuals (57.4% were males, 42.6% females) were included in this study. Mean age was 65.03 ± 18.67 years, while the age range was 19–96 years with a median of 68 years. Mechanical ventilated were among 33.1% of the participants, while 41.8% had nasogastric feeding device.

Clinical data was available only for 144 (91.1%) patients due to difficulties with the clinical examination. 12.7% of all participants had partial or full dentures. One third (31.3%) of the patients were total edentulous, from them only 28.9% had a denture. Fourteen percent of the study population presented Angular Cheilitis. All participants consumed chronic medicines. The common prescriptions were: Clonazepam (30.1%), which is a benzodiazepine drug having anxiolytic, anticonvulsant, muscle relaxant and sedative properties and Lactulose (17.0%), which is a synthetic, non-digestible sugar, used in the treatment of chronic constipation and hepatic encephalopathy. There were multiple routes of drug administration depending on the subject’s health condition.

The mean number of residual teeth was 11.35 ± 10.77. Age was found to be significantly correlated in a decreasing relationship with number of residual teeth (Pearson coefficient: 0.510, *P* < 0.001). Mean number of caries cavitation was 4.17 ± 4.50. Table 1 presents the associations by gender, Clonazepam obtaining with dichotomy oral hygiene (OH) scores (OHI2) and denture usage. Gender was not associated to OH scores and denture usage. Most of the patients (75%) who received Clonazepam presented higher OH score (*P* = 0.018). Obtaining Clonazepam was not associated with denture usage.

**Table 1:** Distribution of dichotomic plaque scores and denture usage by gender and medical status.

		OHI2						*P* ^*^	Denture						*P* ^*^
		0 (0–2)		1 (3)		Total			0 no		1 yes		Total		
		*n*	%	*n*	%	*n*	%		*n*	%	*n*	%	*n*	%	
**Gender**	Male	28	51.9	26	48.1	54	57.4	0.061	73	90.1	8	9.9	81	56.6	0.264
Female	13	32.5	27	67.5	40	42.6	52		83.9	10	16.1	62	43.4	
**Clonazepam**	No	34	51.5	32	48.4	66	70.2	0.018^*^	86	86.0	14	14.0	100	69.9	0.437
Yes	7	25.0	21	75.0	28	29.8	39		90.7	4	9.3	43	30.1	

**Notes.**

* Pearson Chi square, statistically significance at *P* < 0.05.

The number of caries cavitations, number of residual teeth and carious teeth percentage were not found to be statistical significant among patients who were being mechanically ventilated. Number of residual teeth in the mouth were significantly lower when using a nasogastric feeding device (8.57 ± 10.43 vs. 13.40 ± 10.62, *P* = 0.007). Table 2 presents the numerical descriptive results of the number of caries cavitations, number of residual teeth, and carious teeth percentage by gender, OHI2 score and Clonazepam obtaining. Females had significantly higher number of caries cavitation than men (5.25 ± 5.25 and 3.36 ± 3.70 respectively, *P* = 0.044). The number of caries cavitation was higher among patients with higher OHI2 scores (5.96 ± 5.12 vs. 2.00 ± 2.23, *P* < 0.001) and when taking Clonazepam (5.89 ± 5.71 vs. 3.47 ± 3.73, *P* = 0.018). Number of residual teeth was higher in the low OH score group (20.98 ± 7.75 vs. 14.23 ± 8.62, *P* < 0.001). Carious teeth percentage was higher among the high OH score group (53.61 ± 35.03 vs. 14.88 ± 23.00, *P* < 0.001).

**Table 2:** Mean and standard deviation of number of caries cavitations, number of residual teeth, and percentages of carious teeth by gender, dichotomic plaque scores, and taking Clonazepam.

		Number of caries cavitations				Number of residual teeth				Carious teeth percentage			
		*N*	Mean ± SD	CI	*P* ^*^	*N*	Mean ± SD	CI	*P* ^*^	*N*	Mean ± SD	CI	*P* ^*^
**Gender**	Male	53	3.36 ± 3.70	2.34–4.38	0.044^*^	81	11.58 ± 10.64	9.23 ± 13.93	0.776	53	31.62 ± 34.17	22.20–41.04	0.113
Female	40	5.25 ± 5.25	3.57–6.93	63		11.06 ± 11.02	8.29 ± 13.84	40		43.42 ± 36.48	31.75–55.08
**OHI2**	0 (0–2)	40	2.00 ± 2.23	1.29–2.71	<0.001^*^	41	20.98 ± 7.75	18.53–23.42	<0.001^*^	40	14.88 ± 23.00	7.53–22.24	<0.001^*^
1 (3)	51	5.96 ± 5.12	4.52–7.40	53		14.23 ± 8.62	11.85 ± 16.60	51	53.61 ± 35.03	43.76–63.46			
**Clonazepam**	No	66	3.47 ± 3.73	2.55–4.39	0.018^*^	100	11.67 ± 10.97	9.49–13.85	0.597	66	32.96 ± 33.83	24.64–41.27	0.112
Yes	27	5.89 ± 5.71	3.63–8.15	44		10.64 ± 10.38	7.48–13.79	27		45.84–38.33	30.68–61.00		

**Notes.**

* Independent *t* test, statistical significance at *P* < 0.05.

The results of the multivariate logistic regression analysis indicated that only the percentage of carious teeth was a predictor for a high OH score (OR = 1.05, *P* = 0.002, *R*^2^ = 0.435) (Table 3). In a linear regression analysis, high OH score was a predictor for caries cavitation (*P* < 0.001), number of residual teeth (*P* < 0.001), and for carious teeth percentage (*P* < 0.001, Table 4).

**Table 3:** Logistic regression for effect of independent variables on dichotomic plaque score group.

	B	OR	95% CI	*p*
Gender	−0.36	0.70	0.24–2.06	0.516
Clonazepam	−0.90	0.41	0.12–1.32	0.134
Number of teeth	−0.00	0.98	0.92–1.08	0.950
Percentages caries	0.05	1.05	1.02–1.08	0.002^*^
Constant	−0.19	0.83	—	0.882

**Notes.**

* Nagelkerke *R*^2^ = 0.435.

**Table 4:** Linear regression for effect of independent variables on number of caries cavitations.

	B	Beta	95% CI	*P*
**Outcome: number of caries cavitations**				
Caries cavitations (-response)	1.06	0.12	−0.70–2.82	0.234
Dichotomic plaque score (OHI2)	3.80	0.42	2.07–5.53	<0.001
Mech ventilation	1.52	0.15	−0.42–3.47	0.124
Constant	0.51	—	−1.43–2.46	0.601
**Outcome: number of residual teeth**				
Age	−0.94	−0.19	−0.20–0.01	0.076
Dichotomic plaque score (OHI2)	−6.33	−0.36	−9.83–−2.83	0.001
Nasogastric tube	1.08	0.57	−2.86–5.03	0.587
Mech ventilation	3.45	0.18	−0.68–7.59	0.100
Clonazepam	0.08	0.00	−3.80–31.34	0.967
Constant	23.40	—	15.47–31.34	<0.001
**Outcome: percentage of carious teeth**				
Age	0.27	0.17	−0.10–0.64	0.148
Gender	6.80	0.09	−6.54–20.14	0.314
Dichotomic plaque score (OHI2)	35.69	0.50	22.15–49.22	<0.001
Clonazepam	5.60	0.07	−9.27–20.47	0.456
Constant	−3.67	—	−28.18–20.84	0.767

## Discussion

The survey included hospitalized individuals with a mean age of more than 65 years, which is younger than in previous published studies, since the inclusion criteria enabled participation for individuals older than 18 years (Peltola, Vehkalahti & Wuolijoki-Saaristo, 2004; Simunković et al., 2005; Iglesias Corchero & García Cepeda, 2008). This wide age range may explain the difference in the clinical findings. The literature displays edentulous prevalence of 20–45.3% in a variety of studies (Eachempati et al., 2013; Lo, Yan & Dyson, 2004; Peltola, Vehkalahti & Wuolijoki-Saaristo, 2004; Simunković et al., 2005). The WHO published comprehensive study presenting major differences between countries in prevalence of edentulousness among elderlies (Petersen & Yamamoto, 2005). Additionally, the number of residual teeth (a mean of 11.35) decreased with increasing age, in spite of the wide age range and many young participants, lower number than the results found in previous studies (12.4 in Peltola, Vehkalahti & Wuolijoki-Saaristo, 2004, in Finland, 12.9 in Arpin, Brodeur & Corbeil, 2008, in Canada). A possible explanation for this gap may be worse caries scores in Israel, comparing similar age groups in Finnish and Canadian populations (Zusman et al., 2005; World Health Organization, 2000; Sheiham & Sabbah, 2010). High caries prevalence may lead to higher potential of tooth loss and eventually, more edentulousness.

Interestingly, in our study only very few participants (12.7%) had any kind of dentures. 71.1% of the total edentulous participants did not have dentures. This finding is relatively high, comparing to previous studies (as 18% in Peltola, Vehkalahti & Wuolijoki-Saaristo, 2004, and 21% in Iglesias Corchero & García Cepeda, 2008), but we must be cautious interpreting these results.

Mean number of decayed teeth in our study was 4.17 teeth, and 82.8% were with untreated caries. Arpin et al. found only 1.62 teeth, with 49.3% of elderly with untreated caries (Arpin, Brodeur & Corbeil, 2008). According to the National Health and Nutrition Examination Survey (1999–2004), 23% of 65 year and older seniors have untreated caries and the mean number of decayed teeth was 0.39 teeth for 65–74 year olds and 0.47 for 75 years and above (NIDCR, 2014). Our study showed high prevalence of caries and subsequently many treatment needs among the study population.

In our study, females had higher number of caries cavitations than males. A similar finding was observed among elderly in other study (Hämäläinen et al., 2004) which found that males had more intact teeth and lower DMF scores than women (Hämäläinen et al., 2004).

As expected, we found association between number of caries cavitation and low OH. This phenomenon is well known and is re-established in recent studies (Hashim, Williams & Thomson, 2013; Dawani et al., 2012). We also found a statistically significant association between the number of caries cavitation and taking Clonazepam. Clonazepam is a benzodiazepine with many side effects including Xerostomia, which might explain higher numbers of cavitated teeth (Abdollahi & Radfar, 2003).

Oral hygiene was poor and in line with previous observations among this population (Peltola, Vehkalahti & Wuolijoki-Saaristo, 2004). Several explanations had been given for the neglect of daily oral hygiene in LTC patients. One of the possibilities was that the nursing workers are not qualified to assist the institutionalized elderly with oral care (Peltola, Vehkalahti & Wuolijoki-Saaristo, 2004). The poor oral hygiene status urged us to include oral care protocol for the health care personnel, since the current study was part of a comprehensive community intervention program (Bilder et al., 2011).

Surprisingly, according to the clinical examiner’s impression, very few subjects presented oral mucosal lesions and acute gingivitis. These results were not defined as a direct objective of the study, but the literature presents a high prevalence of oral mucosal lesions among institutionalized elderly (Simunković et al., 2005; Rabiei et al., 2010). A possible explanation for that might be the low percentages (12.6%) from the current study participants who were using their dentures. In addition, most of the participants received anti-inflammatory and/or antibiotic medications. Furthermore, according to the hospital’s infection control protocol, all patients received daily treatment of 0.12% chlorhexidine solutions for cleaning oral cavity. All those treatments may have affected the mucosal and gum health status.

Prevalence of angular cheilitis was found in similar proportions in the literature (Peltola, Vehkalahti & Wuolijoki-Saaristo, 2004) (14% vs. 19%). However, in the general elderly population including the LTC hospitalized patients, prevalence of angular cheilitis occur in a range of 1–5% (Kovac-Kovacic & Skaleric, 2000; Espinoza et al., 2003; Mujica, Rivera & Carrero, 2008). We assume that our finding of relatively high prevalance was influenced by the high number of patients with nasogastric feeding device, but this specific finding must be investigated later.

This study has some limitations that one must take into consideration while interpreting the results: the sample was a convenient sample and not a representative of the LTC institutionalized patients in geriatric and psychiatric hospitals in Israel. The majority of the participants had serious complicated medical conditions and were bound to medical devices such as nasogastric feeding (41.8%) or mechanical ventilation (33.1%), which might influence the findings and would explain differences in the results of various studies. Due to the fact that Hertzog Hospital is one of the biggest hospitals and and one of the leaders in treating special needs patients, it hosts the most complicated geriatric and psychiatric patients in Israel. The oral examination was conducted in the hospital beds with very limited access to the oral cavity, while the patients were not always fully cooperative. As a result, it is recommended to use caution when interpreting the results.

## Conclusion

Oral health among LTC institutionalized patients in a geriatric and psychiatric hospital is poor and the majority of participants had unmet dental treatment needs. Focusing on primary prevention and oral hygiene is recommended by providing daily assistance in oral hygiene procedures. This assistance must be provided by committed health care personnel. More attention should be given to enabling access to dental care among this special population. Further national and international scale studies are needed to expand our knowledge on LTC patients’ dental status and needs. That would allow us to evaluate health care personnel attitudes and performance in order to improve patients’ oral status and quality of life.

## Supplemental Information

10.7717/peerj.423/supp-1 Supplemental Information Data

## References

[ref-1] Abdollahi M, Radfar M (2003). A review of drug-induced oral reactions. Journal of Contemporary Dental Practice.

[ref-2] Abdollahi M, Rahimi R, Radfar M (2008). Current opinion on drug-induced oral reactions: a comprehensive review. Journal of Contemporary Dental Practice.

[ref-3] Abnet CC, Qiao YL, Dawsey SM, Dong ZW, Taylor PR, Mark SD (2005). Tooth loss is associated with increased risk of total death and death from upper gastrointestinal cancer, heart disease, and stroke in a Chinese population-based cohort. International Journal of Epidemiology.

[ref-4] Arpin S, Brodeur JM, Corbeil P (2008). Dental caries, problems perceived and use of services among institutionalized elderly in 3 regions of Quebec, Canada. Journal (Canadian Dental Association).

[ref-5] Berkey DB, Scannapieco FA (2013). Medical considerations relating to the oral health of older adults. Special Care in Dentistry.

[ref-6] Bharti V, Bansal C (2013). Drug-induced gingival overgrowth: the nemesis of gingiva unravelled. Journal of Indian Society of Periodontology.

[ref-7] Bilder L, Zini A, Vered Y, Sgan-Cohen H, Mann J (2011).

[ref-8] Chan M, Lim YP, Ernest A, Tan TL (2010). Nutritional assessment in an Asian nursing home and its association with mortality. The Journal of Nutrition, Health & Aging.

[ref-9] Dawani N, Nisar N, Khan N, Syed S, Tanweer N (2012). Prevalence and factors related to dental caries among pre-school children of Saddar town, Karachi, Pakistan: a cross-sectional study. BMC Oral Health.

[ref-10] Eachempati P, Shenoy VK, Jain N, Singh S (2013). Prosthodontic status and needs of elderly institutionalized residents in Mangalore: a prospective study. Indian Journal of Dental Research.

[ref-11] Espinoza I, Rojas R, Aranda W, Gamonal J (2003). Prevalence of oral mucosal lesions in elderly people in Santiago, Chile. Journal of Oral Pathology and Medicine.

[ref-12] Hämäläinen P, Meurman JH, Keskinen M, Heikkinen E (2004). Changes in dental status over 10 years in 80-year-old people: a prospective cohort study. Community Dentistry and Oral Epidemiology.

[ref-13] Hashim R, Williams S, Thomson WM (2013). Oral hygiene and dental caries in 5- to 6-year-old children in Ajman, United Arab Emirates. International Journal of Dental Hygiene.

[ref-14] Iglesias Corchero AM, García Cepeda JR (2008). Oral health in people over 64 years of age, institutionalized in Centres for the Aged in the Vigo Health District Spain, 2005. Medicina Oral Patologia Oral y Cirugia Bucal.

[ref-15] Isaksson R, Söderfeldt B (2007). Oral status and treatment needs among elderly within municipal long-term care 2002–2004. Swedish Dental Journal.

[ref-16] Kaiser MJ, Bauer JM, Rämsch C, Uter W, Guigoz Y, Cederholm T, Thomas DR, Anthony PS, Charlton KE, Maggio M, Tsai AC, Vellas B, Sieber CC (2010). Mini Nutritional Assessment International Group Frequency of malnutrition in older adults: a multinational perspective using the mini nutritional assessment. Journal of the American Geriatrics Society.

[ref-17] Kovac-Kovacic M, Skaleric U (2000). The prevalence of oral mucosal lesions in a population in Ljubljana, Slovenia. Journal of Oral Pathology and Medicine.

[ref-18] Kozyrskyi A, De Coster C, St John P (2000). http://mchp-appserv.cpe.umanitoba.ca/reference/longstay.pdf.

[ref-19] Lo EC, Yan L, Dyson JE (2004). Oral health status of institutionalized elderly in Hong Kong. Community Dental Health.

[ref-20] Matear DW (1999). Demonstrating the need for oral health education in geriatric institutions. Probe.

[ref-21] Mujica V, Rivera H, Carrero M (2008). Prevalence of oral soft tissue lesions in an elderly Venezuelan population. Medicina Oral Patologia Oral y Cirugia Bucal.

[ref-22] National Institute of Dental and Craniofacial Research (2000). http://www.nidcr.nih.gov/DataStatistics/SurgeonGeneral/sgr/.

[ref-23] NIDCR (2014). http://www.nidcr.nih.gov/DataStatistics/FindDataByTopic/DentalCaries/DentalCariesSeniors65older.htm.

[ref-24] Niessen LC (2000). Geriatric dentistry in the next millennium: opportunities for leadership in oral health. Gerodontology.

[ref-25] Osterberg T1, Carlsson GE, Sundh V, Mellström D (2008). Number of teeth—a predictor of mortality in 70-year-old subjects. Community Dentistry and Oral Epidemiology.

[ref-26] Peltola P, Vehkalahti MM, Wuolijoki-Saaristo K (2004). Oral health and treatment needs of the long-term hospitalised elderly. Gerodontology.

[ref-27] Petersen PE, Bourgeois D, Ogawa H, Estupinan-Day S, Ndiaye C (2005). The global burden of oral diseases and risks to oral health. Bulletin of the World Health Organization.

[ref-28] Petersen PE, Yamamoto T (2005). Improving the oral health of older people: the approach of the WHO Global Oral Health Programme. Community Dentistry and Oral Epidemiology.

[ref-29] Rabiei M, Kasemnezhad E, Masoudi rad H, Shakiba M, Pourkay H (2010). Prevalence of oral and dental disorders in institutionalised elderly people in Rasht, Iran. Gerodontology.

[ref-30] Saletti A1, Johansson L, Yifter-Lindgren E, Wissing U, Osterberg K, Cederholm T (2005). Nutritional status and a 3-year follow-up in elderly receiving support at home. Gerontology.

[ref-31] Samaranayake LP, Wilkieson CA, Lamey PJ, MacFarlane TW (1995). Oral disease in the elderly in long-term hospital care. Oral Diseases.

[ref-32] Schmid H (2009). http://www.euro.centre.org/data/1254227500_87459.pdf.

[ref-33] Sheiham A (2005). Oral health, general health and quality of life. Bulletin of the World Health Organization.

[ref-34] Sheiham A, Sabbah W (2010). Using universal patterns of caries for planning and evaluating dental care. Caries Research.

[ref-35] Silness J, Loe H (1964). Periodontal disease in pregnancy. II. Correlation between oral hygiene and periodontal condition. ACTA Odontologica Scandinavica.

[ref-36] Simunković SK, Boras VV, Pandurić J, Zilić IA (2005). Oral health among institutionalised elderly in Zagreb, Croatia. Gerodontology.

[ref-37] World Health Organization (2000). http://whqlibdoc.who.int/hq/2000/WHO_NMH_MNC_ORH_Caries.12y.00.3.pdf.

[ref-38] World Health Organization (2013). Oral health surveys. Basic methods.

[ref-39] Wyatt CC (2002a). Elderly Canadians residing in long-term care hospitals: part I. Medical and dental status. Journal (Canadian Dental Association).

[ref-40] Wyatt CC (2002b). Elderly Canadians residing in long-term care hospitals: part II. Dental caries status. Journal (Canadian Dental Association).

[ref-41] Zusman SP, Ramon T, Natapov L, Kooby E (2005). Dental health of 12-year-olds in Israel-2002. Community Dental Health.

